# Stopping the aged brain from eating itself

**DOI:** 10.18632/aging.205887

**Published:** 2024-05-07

**Authors:** Mar Puigdellívol, Guy C. Brown

**Affiliations:** 1 Departament de Biomedicina, Facultat de Medicina, Institut de Neurociències, Universitat de Barcelona, Spain; 2 Institut d'Investigacions Biomèdiques August Pi i Sunyer (IDIBAPS), Spain; 3 Department of Biochemistry, University of Cambridge, United Kingdom

**Keywords:** aging, synapses, phagocytosis, neuroinflammation, P2Y receptor _6_, microglia

The brain shrinks with age, accompanied by a loss of synapses and memory. We outline here recent evidence in mice that this loss is due to microglial phagocytosis of the synapses, mediated by the microglial P2Y_6_ receptor (P2Y_6_R). 

Brain atrophy during aging appears to be partly due to brain cells, called microglia, eating bits of neurons and the connections between neurons, called synapses. Brain shrinkage and loss of synapses correlate with age-associated memory impairment [1], which affects 50% of people over 60 years old, causing reduced well-being, mental function, and economic activity [2]. There is evidence in mice that aging-induced loss of synapses and memory is due to the phagocytosis (i.e. eating) of synapses by microglia [3, 4]. Microglial phagocytosis is regulated by several factors, including the microglial P2Y_6_ receptor (P2Y_6_R) activated by extracellular UDP (uridine diphosphate) [5, 6]. We recently reported that microglial phagocytosis of synapses during aging is mediated by P2Y_6_R [7]. Inhibition or knockout of P2Y_6_R reduced microglial phagocytosis of synapses and synaptic loss in co-cultures of neurons and microglia. *In vivo*, microglial phagocytosis of synapses was increased in the brains of aged (17 months old) wild-type mice, compared to adult (4 months old) mice, but this increase was absent in P2Y_6_R knockout mice. P2Y_6_R knockout mice also had reduced aging-associated loss of synapses and memory [7]. Thus, inhibiting P2Y_6_R can reduce the loss of synapses and memory with age in mice, probably by preventing microglial phagocytosis of synapses (Figure 1).

**Figure 1:**
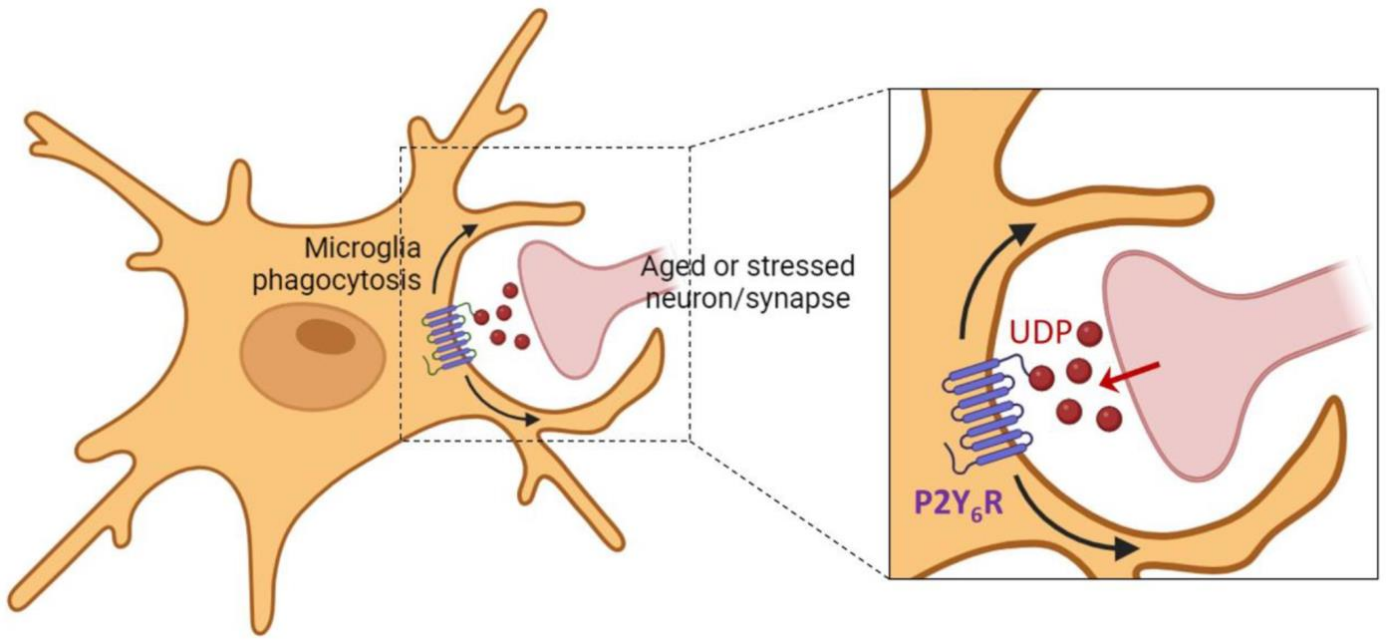
**Microglial phagocytosis of synapses induced by UDP.** Aged and/or stressed synapses and neurons may release UDP that activates the microglial P2Y_6_ receptor, inducing microglial phagocytosis of the synapse or neuron. The image is created with BioRender.

Neurons in the aged brain commonly contain aggregates of the protein tau, referred to as ‘primary age-related tauopathy’ (PART), and we have previously reported that transgenic mice with neuronal tauopathy (P301S *MAPT* mice) had neuronal loss and memory deficits that were reduced by crossing with P2Y_6_R knockout mice [8]. Tauopathy is particularly important in Alzheimer’s disease, which is also characterized by extracellular aggregates of the protein amyloid beta (Aβ) and extensive loss of neurons and memory. We found that P2Y_6_R knockout prevented Aβ-induced microglial phagocytosis of neurons and reduced Aβ-induced memory loss in mice [8]. P2Y_6_R knockout or inhibition also prevented neuronal loss induced by the inflammatory stimulus lipopolysaccharide [6, 9], relevant to aging because brain inflammation increases with age [4]. Brain seizures also increase in the aged, as a result of multiple pathologies, and it was recently shown that brain-seizure-induced loss of neurons and memory was reduced in P2Y_6_R knockout mice as a result of reduced microglial phagocytosis of neurons [10]. Thus, P2Y_6_R-dependent microglial phagocytosis may contribute to the pathology of Alzheimer’s disease, neuroinflammation and seizures, as well as normal aging.

Microglial phagocytosis of synapses also occurs during development, but in this case is beneficial by shaping neuronal networks according to experience. We found that young P2Y_6_R knockout mice had reduced microglial phagocytosis of synapses and reduced memory [11], indicating that P2Y_6_R contributes to microglial phagocytosis of synapses during development. It is unclear whether P2Y_6_R affects memory in the ‘healthy’ adult, but P2Y_6_R knockout mice retain memory better with age [7].

What is inducing microglial phagocytosis of the brain in aging? We do not know for sure, but some factors that accumulate with age (such as Aβ aggregates, tau aggregates, or excess glutamate) stress neurons such that they expose so-called “eat-me” signals (such as UDP) that induce microglia to eat the neurons. Additionally, there is a general increase in inflammation within the brain with age that activates microglia and stimulates microglial phagocytosis [4], in part by the release of ‘opsonins’, such as complement factors C1q and C3, that bind to neurons and synapses, inducing microglia to phagocytose them [3, 4]. UDP activation of P2Y_6_R induces the engulfment phase of microglial phagocytosis [5], and expression of the receptor is increased by inflammation, while excitation of neurons and stress of other cells induces UDP release [5, 10].

Overall, we know that P2Y_6_R regulates microglial phagocytosis and this can contribute to the loss of synapses, neurons, and memory with age and age-related pathologies in mice. We do not know whether P2Y_6_R does the same in humans and whether inhibition of P2Y_6_R can reverse age-associated memory loss, but it would be important to find out.

## References

[1] Morrison JH, et al. Nat Rev Neurosci. 2012; 13:240–50. 10.1038/nrn3200 22395804 PMC3592200

[2] Larrabee GJ, et al. Int Psychogeriatr. 1994; 6:95–104. 10.1017/s1041610294001663 8054499

[3] Shi Q, et al. J Neurosci. 2015; 35:13029–42. 10.1523/JNEUROSCI.1698-15.2015 26400934 PMC6605437

[4] Antignano I, et al. Cell Mol Life Sci. 2023; 80:126. 10.1007/s00018-023-04775-y 37081238 PMC10119228

[5] Koizumi S, et al. Nature. 2007; 446:1091–5. 10.1038/nature05704 17410128 PMC3464483

[6] Neher JJ, et al. Glia. 2014; 62:1463–75. 10.1002/glia.22693 24838858 PMC4336556

[7] Dundee JM, et al. Aging Cell. 2023; 22:e13761. 10.1111/acel.13761 36565471 PMC9924939

[8] Puigdellívol M, et al. Cell Rep. 2021; 37:110148. 10.1016/j.celrep.2021.110148 34965424 PMC8733854

[9] Milde S, et al. J Neuroinflammation. 2021; 18:225. 10.1186/s12974-021-02280-2 34635136 PMC8504061

[10] Umpierre AD, et al. Neuron. 2024. [Epub ahead of print]. 10.1016/j.neuron.2024.03.017 38614103 PMC11189754

[11] Dundee JM, et al. J Neurosci. 2023; 43:8090–103. 10.1523/JNEUROSCI.1089-23.2023 37758475 PMC10697425

